# Xerophthalmia in Picky Eater Children

**DOI:** 10.7759/cureus.22846

**Published:** 2022-03-04

**Authors:** Aiman Ifwat, On Heong Liew, Hanisah Abdul Hamid, Sylves Patrick, Shuaibah Ab.Ghani

**Affiliations:** 1 Department of Ophthalmology, Hospital Queen Elizabeth, Kota Kinabalu, MYS; 2 Department of Ophthalmology, Sabah Women and Children Hospital, Kota Kinabalu, MYS; 3 Department of Ophthalmology, Faculty of Medicine and Health Sciences, Universiti Malaysia Sabah, Kota Kinabalu, MYS

**Keywords:** bitot’s spots, corneal xerosis, conjunctival xerosis, vad, xerophthalmia

## Abstract

Xeropthalmia refers to a range of ocular symptoms caused by vitamin A deficiency (VAD), ranging from night blindness and Bitot’s spots to corneal xerosis, ulceration, and keratomalacia, which can lead to blindness. We report two cases of xerophthalmia in children with intellectual disabilities. Ocular examination revealed generalized conjunctival xerosis, corneal xerosis, and dense superficial punctate keratopathy. Both share a history of a strict self-selective diet of mainly rice and noodles. Serum vitamin A levels for these children showed a very low level (<0.10 µmol/L) and were categorized as severe VAD. One of the cases showed signs of improvement, and the other one succumbed to death secondary to pneumonia. Therefore, proper history-taking, early detection, and prompt treatment are important to prevent the devastating sequelae of VAD.

## Introduction

Xeropthalmia is a wide range of ocular symptoms due to vitamin A deficiency (VAD). Night blindness and Bitot’s spots are common ocular signs of VAD, as are corneal xerosis, ulceration, and keratomalacia, which lead to blindness [1]. Although the daily need for vitamin A is considered a minute, its deficiency is still a global health problem. Taking this into account, it is of utmost importance to identify the clinical signs and symptoms related to VAD for early detection and prompt treatment commencement. Herein, we report two cases of xerophthalmia in two intellectually disabled children of school-going age.

## Case presentation

Case 1

**Figure 1:**
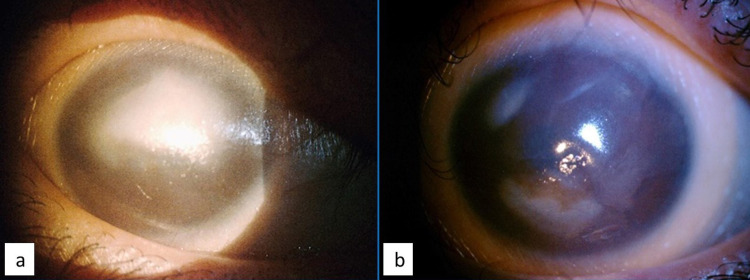
Generalized conjunctival xerosis and corneal xerosis with cornea scarring (a: right eye, b: left eye).

A 12-year-old male with Down’s syndrome appeared with red eyes that were gradually becoming worse and whitish opacity on the cornea in both eyes (OU). There was no history of ocular trauma and no complaint of reduced vision in a bright or poorly lit environment. Visual acuity elicited perception of light in both eyes with poor cooperation from the child during the examination. Ocular examination revealed the absence of tear film, generalized conjunctival xerosis, corneal xerosis, and dense generalized superficial punctate keratopathy (SPK) with corneal scarring in OU (Figure 1). Further history from the parents revealed that his daily dietary intake consisted mainly of plain fried noodles. The patient’s serum retinol concentration was <0.1 µmol/L (normal range: 0.9-3.0 µmol/L). A diagnosis of xerophthalmia secondary to VAD was established. His parents were advised and encouraged to incorporate foods rich in vitamin A in his diet. He was also prescribed oral multivitamins, topical lubricants, and chloramphenicol ointment. Unfortunately, he succumbed to death due to septic shock with multiple-organ failure secondary to pneumonia.

Case 2

**Figure 2:**
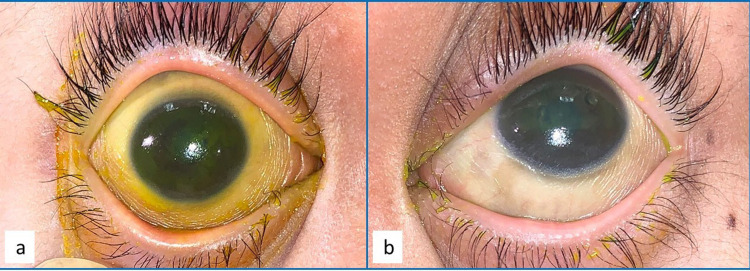
Generalized conjunctival xerosis and corneal xerosis in both eyes (a: right eye, b: left eye).

A 10-year-old male with an autism spectrum disorder presented with intermittent redness for two months in OU. His mother noticed frequent eye rubbing and signs of disturbed vision of three weeks duration prior to the presentation. He had no history of foreign body entry into his eyes, ocular trauma, or known allergies. Visual acuity using Lea Grating corresponded to 6/180 in OU, and again, this was an uncooperative child. On ocular examination, there were generalized conjunctival xerosis and absence of tear film in OU. Both corneas were hazy and had extremely dry surfaces and dense confluent SPK with visible iris details. Corneal scarring and Bitot’s spots were not seen (Figure 2). Further history from the parents revealed that he is a picky eater who only relishes fried rice with soy sauce or noodles for meals. Serum retinol showed a very low level of <0.10 µmol/L (normal range: 0.9-3.0 µmol/L); therefore, a diagnosis of xerophthalmia secondary to VAD was made. A nutritionist provided counseling as part of his management plan, and the necessity of a balanced diet and nutrition in his daily diet was highlighted. He was started on oral multivitamins, preservative-free topical lubricants, and chloramphenicol ointment.

## Discussion

​​​​​​Vitamin A is a fat-soluble vitamin that humans and other vertebrates require. It plays a vital role in cell development, metabolism, immune function, vision, and reproductive function [2]. Particularly in the eye, vitamin A plays a vital role in the retinal pigment epithelium’s visual cycle. It also takes an important role in the growth of the epithelium and the differentiation of the limbal stem cells on the eye’s surface [3]. Fish oil, leafy green vegetables, carrots, and apricots are natural sources of vitamin A, whereas cod liver oil and beef or turkey liver are the foods richest in vitamin A.

Xerophthalmia is a progressive ocular disease caused by VAD. It can occur to people of any age group; however, it is more common in preschoolers, adolescents, and pregnant women. Children, on the other hand, are more susceptible to VAD and xerophthalmia due to their higher vitamin A requirements for growth. Furthermore, children are more susceptible to intestinal infestations and infections, which can decrease vitamin A absorption [4].

The World Health Organization (WHO) estimates that around 228 million children worldwide have VAD, causing 5-10 million cases of eye disease and 1-3 million deaths. Xerophthalmia becomes the leading cause of blindness in children worldwide, with the estimation of a quarter to half a million blind children a year [5]. It is more common in developing countries and impoverished regions [6]; however, there are still reported cases of xerophthalmia in developed countries [7,8]. The prevalence is higher in South East Asia and Africa [5].

In a survey conducted by the Ministry of Health of Malaysia and UNICEF in 2002, the result showed that the blood retinol levels in more than 400 children under the age of five years were 0.7 µmol/L in 2.5% of male children and 4.5% of female children. Based on these findings, Malaysian children may be said to have mild subclinical VAD [9]. However, a decade later, the results of two nationwide studies, the South East Asian Nutrition Survey (SEANUTS) of Malaysian children and the MyBreakfast study, revealed that the situation has greatly improved. The SEANUTS Malaysia reported that only a small percentage of children exhibited low Hb (6.6%), serum ferritin (4.4%), or vitamin A levels (4.4%), whereas the MyBreakfast research revealed that more than 50% of the school-aged children received at least 80% of the recommended nutrient intake (RNI) for vitamin A, vitamin C, niacin, riboflavin, thiamine, and iron [10,11]. In other words, VAD is not a major health issue in Malaysia.

Vitamin A is crucial for mucin-producing conjunctival goblet cells. Their dysfunction can lead to the development of severe dry eye disease and associated complications [12]. Therefore, as the disease progresses, the conjunctiva becomes dry with a wrinkled, skin-like appearance known as conjunctival xerosis. This is a reflection of underlying keratinized metaplasia. Bitot’s spots, a unique manifestation of VAD with a dry, triangular white foamy appearance that is usually near the temporal conjunctiva, may be present in addition to conjunctival xerosis [13]. If VAD persists, corneal xerosis may develop, resulting in a cloudy cornea, ulceration, or keratomalacia, in which part or all of the cornea is liquefied [14]. However, these findings may not occur according to the sequence or classification.

A patient is at risk of corneal infections due to the loss of a natural barrier in addition to dry eye syndrome. The reduced sensitivity of the injured cornea often masks subjective discomfort. The presence of corneal scars is not regarded as part of active VAD but rather as a result of a previous bout of the deficiency [14]. Long-term VAD increases the risk of infection-related morbidity and mortality, as well as the risk of blindness [15].

Night blindness is generally the earliest clinical manifestation of VAD and is both a sensitive and specific sign of low serum retinol levels [16,17]. Night blindness occurs because of the progressive inhibition of rhodopsin synthesis on a microscopic level, as vitamin A is a precursor for rhodopsin formation [12]. When serum retinol concentrations fall below 1.0 µmol/L, impaired adaptation to the dark can begin, although it is more common when they fall below 0.7 µmol/L [4]. Many studies have employed a history of night blindness given by parents as part of the method for assessing the prevalence of this condition [18,19]. Both our patients were intellectually disabled, which hinders the ability of parents to notice the sign. In addition, even for normal children, it is difficult to elicit the sign, and the child may not be aware that they have night blindness. Children with VAD that have ocular signs, such as night blindness, have a higher mortality rate compared to those children with VAD who do not have ocular signs. In addition, the mortality rate increases ninefold if the child has both night blindness and Bitot’s spots [20].

Based on the WHO classification (Table 1), case 1 has a mixed appearance of conjunctival xerosis (X1A), corneal xerosis (X2), and corneal scarring (XS), whereby case 2 presented with conjunctival xerosis (X1A) and corneal xerosis (X2) [16].

**Table 1:** Classification of xerophthalmia by the World Health Organization (WHO).

Class	Ocular signs
XN	Night blindness
X1A	Conjunctival xerosis
X1B	Bitot’s spot
X2	Corneal xerosis
X3A	Corneal ulcer/keratomalacia < 1/3 surface
X3B	Corneal ulcer/keratomalacia ≥ 1/3 surface
XS	Corneal scarring
XF	Xerophthalmic fundus

The parent of a child who presented with signs of xeropthalmia must be questioned with a thorough history-taking, including dietary intake, underlying syndrome or intellectual disability, malabsorption problems, systemic infection, and socioeconomic status, as these are the risks for VAD. There are few reported cases of vitamin A deficiency in autistic children who are only being diagnosed after referral to an ophthalmologist despite proper medical follow-up [21]. The ocular manifestations of xerophthalmia may give clues in making the diagnosis as obtaining history in this particular child is difficult. In our cases, the challenges in the diagnosis were to perform proper ocular examinations and to get a history of night blindness in the past.

Confirmation of VAD is achieved by measuring the serum retinol level. According to the WHO, VAD can be clinically or subclinically identified. Retinol concentrations in the blood are used to diagnose subclinical VAD. In children and adults, a serum retinol concentration of 0.70 μmol/L suggests mild vitamin A deficiency, while a value of 0.35 μmol/L denotes severe VAD [22]. In our cases, both patients were categorized as having severe VAD, with serum retinol of <0.10 µmol/L.

Although xeropthalmia is the commonest cause of childhood blindness worldwide, it is preventable via nutritional measures and reversible with a good visual prognosis once treated early. Treatment aims to restore vitamin A to a normal level. This can be achieved through vitamin A supplementation, vitamin A fortification of food (e.g., fortification of cooking oil/cereal product with vitamin A), and dietary diversification [23]. Referral to a dietitian/nutritionist is important to educate parents regarding the proper diet that the patient should take, especially dietary products rich in vitamin A. For ocular treatment, intense topical lubricants, topical prophylactic antibiotics, or management of perforation should be commenced if indicated [24]. However, the real challenge in the treatment in both of our cases was to persuade the child to consume and adhere strictly to a proper diet.

## Conclusions

Albeit the occurrence of xeropthalmia is rare nowadays, children at risk, including those with restrictive dietary intake and intellectual disabilities, should be investigated early for VAD and prompt commencement of treatment to prevent permanent visual loss.
